# Subchronic Oral Toxicity Study of Genetically Modified Rice Rich in β-Carotene in Wistar Rats

**DOI:** 10.3390/ijerph18115526

**Published:** 2021-05-21

**Authors:** Ying Xia, Shanshan Zuo, Yanhua Zheng, Jin Liu, Wenxiang Yang, Xiaoqiao Tang, Xianghong Ke, Qin Zhuo, Xiaoguang Yang, Yang Li, Bolin Fan

**Affiliations:** 1 Hubei Provincial Key Laboratory for Applied Toxicology, Hubei Provincial Center for Disease Control and Prevention, Wuhan 430079, China; kikixy516@163.com (Y.X.); zuoshanshan@whu.edu.cn (S.Z.); zyh512995587@163.com (Y.Z.); lj1070268635@163.com (J.L.); ywx_21@163.com (W.Y.); melon_qiao@163.com (X.T.); kexianghong724@126.com (X.K.); liyang_2021a@163.com (Y.L.); 2 Qingdao Municipal Center for Disease Control and Prevention, Qingdao 266033, China; 3 Key Laboratory of Trace Element Nutrition of National Health Commission (NHC), National Institute for Nutrition and Health, Chinese Center for Disease Control and Prevention, Beijing 100050, China; zhuoqin@ninh.chinacdc.cn (Q.Z.); xgyangcdc@163.com (X.Y.)

**Keywords:** β-carotene, genetically modified rice, safety evaluation, rats

## Abstract

(1) Background: a hybrid black rice rich in β-carotene carrying the *psy* and *crtI* genes (HJM) was evaluated in Wistar rats by a 90-day feeding study, aiming to assess its dietary safety. (2) Methods: the HJM rice and its parental line HS were included in rats’ diets at levels of 73.5% and 75.5%, respectively. The AIN-93 diet was administered as a nutritional control. No adverse effects on animal behavior or weight gain were observed during the study. Blood samples were collected and analyzed, and standard hematological and biochemical parameters were compared. (3) Results: Some parameters were found to be significantly different, though they remained within the normal range for rats of this breed and age. In addition, upon sacrifice, various organs were weighed, and macroscopic and histopathological examinations were performed, with only minor changes to report. (4) Conclusions: HJM rice exhibited no adverse or toxic effects in Wistar rats in this 90-day study.

## 1. Introduction

Rice is one of the most important crops, serving as the staple food for nearly half of the global population [1]. In Asia, where more than 90% of the world’s rice is grown and consumed, at least 30% of the daily caloric intake is from rice. China is the largest rice consumer in the world, accounting for 30% of the world rice consumption [2]. However, the endosperm, the remaining comestible part of rice grains after milling, lacks several essential nutrients, especially provitamin A [3]. Accordingly, a diet consisting predominantly of rice leads to vitamin A deficiency. 

Vitamin A deficiency is a serious public health nutrition concern and has various unfavorable effects on the human body, such as permanent blindness, impairment of the immune system, and exacerbation of infectious diseases [4]. Globally, Vitamin A deficiency is estimated to affect over 190 million preschool children and 19 million pregnant women, and improved nutrition could prevent 1 million to 2 million deaths annually among children [5].

Due to the rapid development of recombinant technologies, success has been obtained in the supplementation of rice with provitamin A. “Golden rice” was first engineered with the aim of increasing the β-carotene content in plants through the biosynthetic pathway [3]. Based on that, optimization of *Erwinia uredovora* carotene desaturase was employed to improve the accumulation of β-carotene content from 1.6 μg/g to 37 μg/g in rice [6].

In this toxicology study, a transgenic rice line (HJM) rich in β-carotene and its parental line (HS) were compared. The phytoene synthase (*psy*) and bacterial phytoene desaturase (*crtI*) genes derived from maize and *Erwinia uredovora* were introduced into the germline of Kongyu131 rice to produce the line GRH, whose rice grains contain β-carotene [7]. The hybrid black rice line HJM was created by crossing GRH with a conventional non-transgenic rice (HS), which has an established history of safe use in China [8].

The safety assessment of transgenic crops is based on the principle of substantial equivalence that has been originally proposed by the OECD [9]. This principle focuses on comparing a transgenic crop to its non-transgenic counterpart using compositional analysis to determine whether the insertion of a gene leads to an imbalance of nutrition constituents. Additionally, animal feeding tests are recommended, as transgenic crops subjected to equivalence analysis are not considered utterly safe and may cause unpredictable changes. The validity of tests evaluating the adverse effects of transgenic crops has been demonstrated [10].

Up to now, almost no harmful effects have been observed for transgenic products including rice [11,12,13,14], maize [15,16], and soybean [17,18]. Nevertheless, few studies have reported the dietary safety assessment of transgenic rice rich in β-carotene. Therefore, in this study, we performed a subchronic feeding experiment to examine the potential adverse effects of the transgenic rice HJM rich in β-carotene.

## 2. Materials and Methods

### 2.1. Test Materials

**Figure 1:**
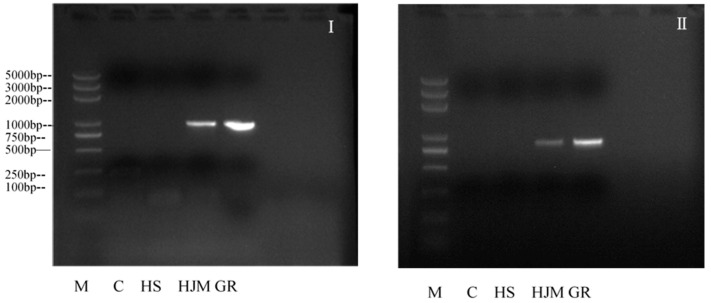
Te Detection of the *psy* gene and the *crtI* gene in HJM (M: 2 kb DNA Marker; C: AIN-93G control diet; HS: non-transgenic parental HS rice; HJM: transgenic HJM rice; GR: golden rice. (**I**): *psy* gene; (**II**): *crtI* gene.).

Transgenic rice (HJM) and its parental counterpart (HS) were obtained from Huazhong Agricultural University, Wuhan, China, and grown under the same conditions and management in Hainan, China. As indicated by the results of the PCR test, the *psy* gene and the *crtI* gene were present in the HJM rice, while not detected in the HS rice (Figure 1). The nutritional analysis of HJM and HS are presented in Table 1.

### 2.2. Diet Formulation and Feeding

After comparing the contents of rice and standard rat diet, flours of HJM and HS were included into rodent diets at concentrations of 73.5% and 75.5%, which were the largest proportions allowed under the premise of ensuring dietary safety. The AIN93G purified diet were included as a nutritional control. All diets were formulated following AIN93G guidelines [10] and adjusted identically to ensure an adequate supply of macronutrients and vitamins. All diets were processed by Wuhan Wanqianjiaxing Bio technology Co, Ltd. (Wuhan, China), and sampled for nutritional analyses at the Hubei Institute of Product Quality Supervision and Inspection (Wuhan, China). 

### 2.3. Animals and Housing

A total of 180 specific-pathogen-free (SPF) Wistar rats (90 males and 90 females), aged 5 weeks, with an average body weight of 60–90g at the initiation of acclimation, were obtained from the SPF Biotechnology Co., Ltd. (Beijing, China) [license number: SCXK(Beijing) 2016–0002]. All animals were kept pairwise in stainless-steel wire cages at 20–26℃ at a relatively humidity of 40–70% with artificial illumination (fluorescent light) on an approximate 12 h light/dark cycle. All rats were provided food and filtered tap water ad libitum. Animal experiments and housing procedures were carried out in accordance with the laboratory animal administration rules of the Ministry of Science and Technology of the People’s Republic of China. All experimental procedures were examined and approved by the animal Management and Use Committee of Hubei Food and Drug Safety Evaluation Center (approval No. 20192619).

### 2.4. Study Method

Following one week of acclimatization, animals were randomly assigned to three groups, with 60 rats in each group (30 males and 30 females per treatment) according to their mean body weight. All rats were provided the corresponding diets and observed for 13 weeks. The animals were inspected daily for mortality and signs of toxicity. Both body weight and food consumption were measured weekly, and the relative food consumption calculated. During the last week of treatment, rats were fasted at least 12 h prior to blood sample collection. Whole blood was taken from the abdominal aorta and evaluated for white blood cells leukocytes (WBC), erythrocytes (RBC), hemoglobin (HGB), hematocrit (HCT), platelets (PLT) and leukocyte classification including percentage of scilicet lymphocytes (LYMPH), neutrophils (NEUT), eosinophils (EO), monocytes (MONO) and basophil (BASO) with an automatic Hematology Analyzer (Sysmex XT-2000iV, Kobe, Japan). Following centrifugation, the sera were assayed for alanine aminotransferase (ALT), aspartate aminotransferase (AST), total protein (TP), albumin (ALB), alkaline phosphatase (ALP), glucose (GLU), blood urea nitrogen (BUN), creatinine (CREA), cholesterol (CHOL), triglyceride (TG), K^+^, Na^+^, Cl^-^, and Ca^2+^ with a biochemical analyzer (Backman AU-680, America). Prothrombin times (PT) and activated partial thromboplastin time (APTT) were measured with an automatic coagulation analyzer (Sysmex CA-510, Kobe, Japan). Following sacrifice, a detailed gross necropsy was performed. Organs including heart, liver, spleen, kidney, adrenal gland, brain, thymus, uterus, ovary, testis, and epididymis were dissected and weighed. The relative organ weight was expressed as a percentage of the final individual brain weight. Histopathological analyses were carried out in the following organs: cerebrum, cerebellum, pituitary, thyroid, thymus, lungs, heart, liver, spleen, kidneys, adrenal gland, stomach, duodenum, jejunum, ileum, colon, rectum, pancreas, lymph nodes, prostate, testicles, epididymis, ovaries, and uterus. Organs were fixed with buffered formalin, embedded in paraffin, sectioned to approximately 4–6 µm-thick slices, stained with hematoxylin-eosin, and examined under a microscope (BX41-32H02, Olympus Optical Co.Ltd, Tokyo, Japan).

### 2.5. Statistical Analysis

The statistical software “Statistical Product and Service Solutions” (SPSS) v14.0(SPSS Inc, Chicago, IL, USA) was employed to evaluate whether the observed difference was attributable to the consumption of diets containing HJM. A one-way analysis of variance (ANOVA) was applied to evaluate the homogeneity variance, and then Dunnett-t multiple comparison tests were conducted to detect the variables differences between the groups. *p* < 0.05 was in all cases considered significant.

## 3. Results

### 3.1. Diet Nutritional Composition

The diets administered to the three groups were nutritionally balanced, with close nutrition content (Table 2).

### 3.2. Clinical Observations, Body Weight, and Food Consumption

**Figure 2:**
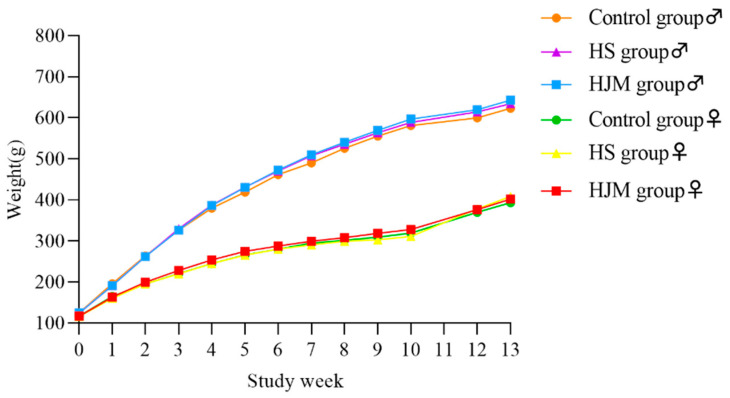
Mean weekly body weight of male and female rats.

Over the course of the study, neither mortality nor adverse effects on animal behavior were observed. Body weights were normal and generally similar in the three groups (Figure 2). 

**Figure 3:**
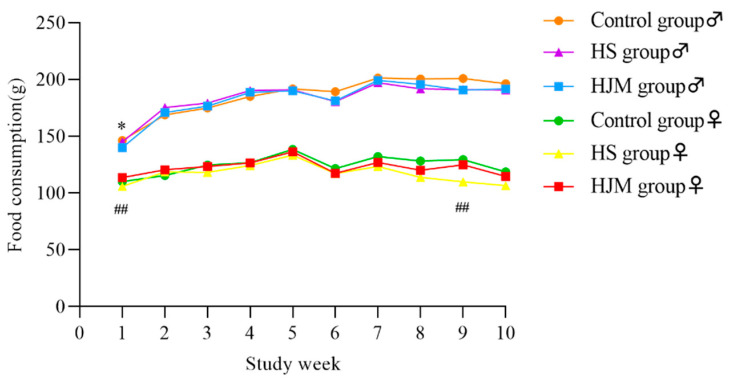
Mean weekly food consumption of male and female rats.

**Figure 4:**
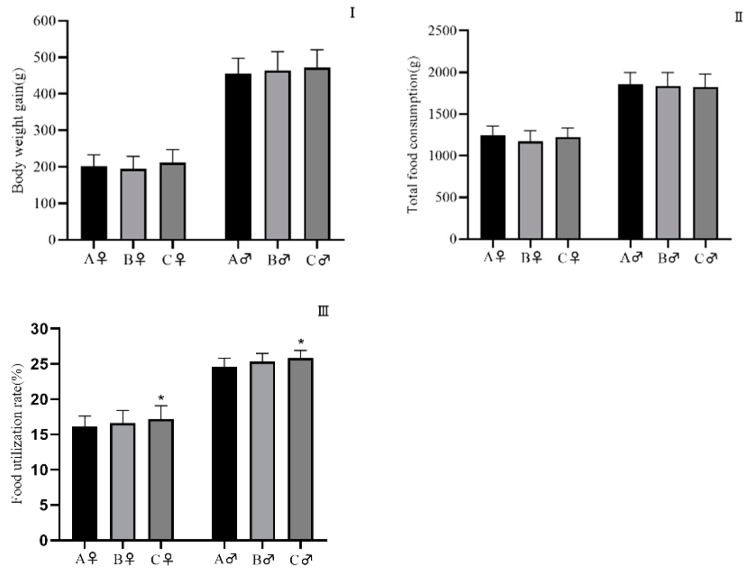
Total body weight gains, total food consumption, and total food utilization for 10 weeks in male and female rats. A: control group; B: HS group; C: HJM group. (**I**): total body weight gain; (**II**): total food consumption; (**III**): total food utilization. Compared with the control group, * *P* < 0.05.

At week 1 and 9, food consumption of the HJM group was higher than the that of the HS group (Figure 3), whereas no change was observed between the male groups. Moreover, there were no differences in total food consumption between the HJM group and the HS group or the control group (Figure 4). The slightly higher total food consumption rate in the HJM group compared with the control group was possibly due to the higher total body weight gain and lower total food consumption of rats fed the HJM diet. Further, no corresponding differences were found between the HJM group and the HS group.

### 3.3. Hematology

Table 3 shows that there were no statistically significant differences in most of the values. The mean MONO% for female rats consuming HJM was slightly higher than for the HS group. However, the difference was not accompanied by differences in other leukocyte classification parameters, nor was it observed in male rats in the three groups.

The mean EO% was lower for male rats of the HJM group compared with those of the control group. The EO% represents a type of leucocytes that, upon infection causing typhoid or paratyphoid fever, could decrease in the blood. This difference was considered a spontaneous alteration, because no other typhoid-oriented related indicators (NEUT%, WBC) were observed. In addition, no statistical difference in EO% was observed between the HJM group and the HS group.

Mean PT was lower in female rats of the HJM group compared with those of the control group. However, no statistical difference in the mean serum APTT level was observed among the three groups, and no equivalent change was observed between the HJM group and the HS group. 

Meanwhile, the values of MONO%, EO%, and PT in the HJM group remained within the normal range. Therefore, these statistical differences were considered to be incidental and of no clinical significance.

### 3.4. Serum Biochemistry

Table 4 shows that the mean ALT and AST values of the HJM group were lower compared with those of the control group. ALT and AST increase in liver cells when liver cells or other tissues are damaged. Nevertheless, the decreased in ALT or AST had no clinical significance. In addition, no statistical difference in ALT and AST was observed between the HJM group and the HS group.

Mean TP and ALB were higher in female rats of the HJM group compared with those of the control group but were significantly decreased in males compared to those of the HS group.

GLU was significantly elevated in females of the HJM group compared with those of the control group, but this difference disappeared when compared to the HS group.

Mean BUN and CREA were lower in female rats compared with those of the control group, but this difference disappeared when compared to the HS group. Besides, the decreased values of BUN and CREA were not considered adverse.

The mean values of CHOL, TG, and Ca^2+^ were higher in female rats of the HJM group compared with the control group. This difference was not considered adverse nor was it related to the consumption of HJM rice, because no statistical difference in mean serum CHOL, TG, and Ca^2+^ levels was observed in female rats between the HJM group and the HS group and no statistical difference was observed in male rats among any of the different treatment groups. 

Moreover, most changes of parameters in HJM-fed rats were still in the normal range and were considered to be not clinically significant. 

### 3.5. Organ/Brain Weight Ratios and Histopathology

**Figure 5:**
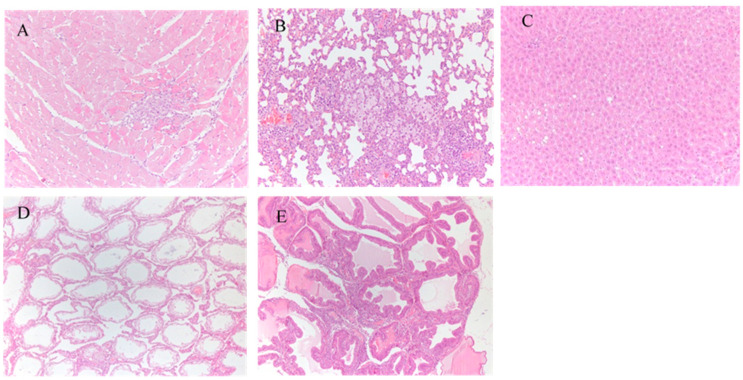
Histopathologic changes in rats in the HJM group. (**A**): inflammatory cell infiltration in the myocardium (HE 10×10); (**B**): pulmonary foam cell formation (HE 10 × 10); (**C**): liver steatosis (HE 10 × 20); (**D**): shrunken testicles (HE 10 × 10); (**E**): inflammatory cell infiltration in prostatic stroma (HE 10 × 10).

A necropsy was conducted on animals, with no gross pathological findings. Table 5 shows the relative organ weights for males and females in the three groups. Compared to the control group, we observed a significant difference in organ/brain weight ratio for the thymus, but there were no statistically significant differences between the HJM and HS groups. Besides, no significant abnormal findings were detected histopathologically in the thymus. The microscopic examinations of the three groups mainly regarded intrahepatic inflammatory cell infiltration, fatty degeneration of the liver, expansion of gastric glands, infiltration of inflammatory cells around the pulmonary artery, and infiltration of inflammatory cells in the myocardium (Figure 5). All of the above spontaneous lesions were comparable among the three groups and common in rats of that age and strain.

## 4. Discussion

The application and development of biotechnology has become a sustainable strategy to improve the quality and quantity of crops so to combat deficiencies in food by enhancing the content of proteins, carbohydrates, lipids, vitamins, and micronutrients [21]. Examples include the “high-lysine rice” to increase lysine content [22] and the “high-amylose rice” to increase resistant-starch content [23]. “Golden rice” is perceived as a mean to increase β-carotene content so to improve the Vitamin A deficiency status of highly populated areas and balance nutritional quality in the human body. Nevertheless, the safety evaluation of these novel foods is a crucial question [20]. Based on the principle of case-by-case analysis, it is essential to carry out strict and comprehensive toxicological assessments on each rice line to confirm its safety as food or feed [24].

At present, substantial equivalence of transgenic crops is required for the approval of commercialization. Based on that, comparison of the nutritional composition between transgenic crops and the parental crops is an important aspect in the safety evaluation of genetically modified food products [25]. In this study, the nutrient compositional content of the HJM rice and its parental HS rice was analyzed, aiming to provide a basis for the design of nutritionally balanced diets for animals. The results showed that there were no differences in nutrition constituents between the two lines and within the ranges reported in the literature, except that the content of active components (β-carotene, procyanidins, and lutein) in HJM rice was significantly higher than in HS rice. Similar consistency in composition was also reported in the study comparing the transgenic rice EH rich in β-carotene and its parental rice Zhonghua 11 [26]. Zhou indicated that a diet containing the high-lysine transgenic rice LR had almost the equivalent nutrients compared with the near-isogenic conventional diet [23]. Although ‘‘substantial equivalence’’ data may provide a theoretical basis for food safety, animal feeding studies are considered more direct and feasible to assess the potential adverse pleiotropic effects that may not be detected in compositional analyses.

Throughout the feeding period, no adverse behavior or clinical effects in either male or female rats were observed. The consumption of HJM rice was higher than that of HS rice in the first and ninth weeks, whereas no significant differences in overall effects were observed, namely, in body weight and total food consumption. In addition, no similar change was observed between males in the groups, indicating that the changes were incidental and irrelevant. A significantly increased total food utilization rate was found in the HJM group compared to the control group, owing to lower total food consumption in the HJM rice group, likely due to the reduced palatability of diets with high rice content.

Some hematology and serum chemistry variables showed statistical difference between rats of the HJM group and the HS group or the control group. Significant different parameters (AST, ALT, TP, ALB) were all related to the liver, but there no differences were observed indicating a liver pathology (weights and histopathology). BUN and CREA values are indicators of kidney function, but the observed decreased values were not considered adverse. GLU, CHOL, TG, and Ca^2+^ were significantly different between rats of the HJM group and those of the control group, but no statistical difference was observed between the HJM group and the HS group. Moreover, no histopathological abnormality was found. Parallel to our study, a few changes in hematological and biochemical indicators were reported also in another study, but were not attributed to the consumption of transgenic rice [12,27].

## 5. Conclusions

In this study, compared with the parental HS rice and the control diet, the transgenic HJM rice exhibited no toxicological effects in Wistar rats. It can be concluded that the transgenic HJM rice rich in β-carotene is as safe as the parental HS rice.

## Figures and Tables

**Table 1:** Nutritional components of HJM and HS.

Nutrients	HJM	HS	Literature Range ^a^
Protein (g/100 g)	9.8	11.7	6.1–9.5
Fat (g/100 g)	2.8	3.1	1.4–5.3
Carbohydrate (g/100 g)	71.9	69.5	57–77
Fiber (%)	0.5	0.5	0.5–6.8
Ash (%)	1.2	1.2	0.9–1.5
Moisture (%)	14.2	14.5	9.1–14.1
Energy (kJ/100 g)	1494	1495	-
Phosphorus (mg/100 g)	289	285	-
Potassium (mg/100 g)	275	272	-
Magnesium (mg/100 g)	97.3	108	-
Calcium (mg/100 g)	12.1	11.1	-
β-carotene (mg/kg)	11.5	0.31	-
Procyanidins (mg/kg)	1396.9	1187.9	-
Lutein (mg/kg)	34.95	22.45	-

^a^ Ranges: from minimum to maximum reported values [12,15,19,20].

**Table 2:** Nutritional composition of diet.

Components (%)	Control Group	HS Group	HJM Group
Crude Protein	17.86	18.01	18.79
Crude Fat	7.0	6.5	6.1
Carbohydrate	64.3	59.2	60.7
Energy, kcal/g	3.77	3.73	3.74

**Table 3:** Hematology parameters of male and female rats (mean ± SD).

	WBC (10^9^/μL)	RBC (10^12^/μL)	HGB (g/L)	HCT (%)	PLT (10^9^/μL)	NEUT %	LYMPH %	MONO %	EO %	BASO %	APTT (s)	PT (s)
Female												
Control group	5.3 ± 1.9	7.59 ± 0.36	149 ± 7	43.8 ± 2.7	787 ± 105	26.7 ± 7.1	70.3 ± 7.2	1.2 ± 0.6	1.8 ± 0.8	0.0 ± 0.0	9.9 ± 1.6	13.5 ± 0.5
HS group	4.6 ± 1.3	7.65 ± 0.44	150 ± 9	44.2 ± 2.6	737 ± 95	24.7 ± 5.3	72.6 ± 5.5	1.0 ± 0.4	1.7 ± 0.6	0.0 ± 0.0	10.1 ± 2.2	13.1 ± 0.6
HJM group	5.2 ± 1.6	7.54 ± 0.42	149 ± 9	43.4 ± 2.2	745 ± 107	25.3 ± 4.6	71.6 ± 5.2	1.3 ± 0.5 ^##^	1.8 ± 0.8	0.0 ± 0.0	10.2 ± 1.9	13.1 ± 0.5 *
Male												
Control group	6.9 ± 1.8	8.20 ± 0.47	148 ± 8	43.1 ± 1.9	875 ± 147	28.4 ± 6.6	69.0 ± 6.9	1.3 ± 0.4	1.3 ± 0.5	0.0 ± 0.0	21.9 ± 7.3	12.8 ± 0.7
HS group	6.0 ± 2.0	8.14 ± 0.55	147 ± 9	43.1 ± 3.0	825 ± 193	29.5 ± 5.0	67.0 ± 5.5	1.3 ± 0.5	2.1 ± 1.2	0.0 ± 0.0	21.4 ± 7.2	12.6 ± 0.8
HJM group	6.5 ± 3.0	8.14 ± 0.46	146 ± 7	42.5 ± 1.9	845 ± 245	30.7 ± 8.8	66.3 ± 8.9	1.2 ± 0.6	1.8 ± 0.7 *	0.0 ± 0.0	21.3 ± 6.5	12.7 ± 0.8

Compared with the control group, * *p* < 0.05; Compared with the HS group, ^##^ *p* < 0.01.

**Table 4:** Serum biochemistry parameters of male and female rats (mean ± SD).

	ALT (U/L)	AST (U/L)	TP (g/L)	ALB (g/L)	ALP (IU/L)	GLU (mmol/L)	BUN (mmol/L)
Female							
Control group	35 ± 10	116 ± 28	59.5 ± 3.7	30.8 ± 2.1	61 ± 26	5.87 ± 1.04	3.86 ± 0.65
HS group	28 ± 6	102 ± 24	62.6 ± 4.1	33.0 ± 2.5	60 ± 18	6.33 ± 1.28	3.19 ± 1.18
HJM group	27 ± 4 **	96 ± 22 **	63.6 ± 3.5 **	33.5 ± 2.1**	60 ± 23	7.27 ± 1.60 **^#^	2.96 ± 0.69 **
Male							
Control group	43 ± 7	141 ± 27	56.6 ± 2.9	28.4 ± 1.3	103 ± 25	6.27 ± 0.78	3.63±0.65
HS group	35 ± 8	132 ± 26	59.4 ± 4.8	29.5 ± 1.9	101 ± 22	6.56 ± 1.60	3.23 ± 0.60
HJM group	37 ± 6 **	132 ± 26	55.8 ± 3.0 ^##^	28.1 ± 1.2 ^##^	94 ± 30	6.81 ± 1.90	3.40 ± 0.40
	**CREA (mmol)**	**CHOL (mmol/L)**	**TG (mmol/L)**	**K^+^ (mmol/L)**	**Na^+^ (mmol/L)**	**Cl^−^ (mmol/L)**	**Ca^2+^ (mmol/L)**
Female							
Control group	78.7 ± 15.4	1.76 ± 0.29	0.71 ± 0.20	4.17 ± 0.22	139.9 ± 1.9	104.7 ± 2.2	2.64 ± 0.08
HS group	61.8 ± 15.9	1.97 ± 0.16	0.88 ± 0.28	4.17 ± 0.25	140.7 ± 2.0	105.1 ± 1.5	2.75 ± 0.10
HJM group	58.3 ± 16.2 **	2.07 ± 0.42 **	0.96 ± 0.52 *	4.08 ± 0.30	139.7 ± 2.0	104.6 ± 1.6	2.74 ± 0.10 **
Male							
Control group	59.5 ± 12.5	1.49 ± 0.38	0.59 ± 0.20	4.60 ± 0.64	140.8 ± 4.2	103.7 ± 2.7	2.39 ± 0.18
HS group	55.5 ± 10.5	1.77 ± 0.40	0.81 ± 0.32	5.08 ± 1.23	142.1 ± 4.3	102.9 ± 3.3	2.52 ± 0.30
HJM group	56.7 ± 7.4	1.56 ± 0.35	0.82 ± 0.53	4.64 ± 0.48	141.4 ± 4.1	103.8 ± 2.3	2.42 ± 0.20

Compared with the control group, * *p* < 0.05, ** *p* < 0.01; Compared with the HS group, ^#^ *p* < 0.05, ^##^ *p* < 0.01.

**Table 5:** Organ/brain weight ratios of male and female rats (mean ± SD).

Heading	Heart	Liver	Spleen	Kidney	Adrenal Gland	Thymus	Uterus/Testis	Ovary/Epididymis
Female								
Control group	0.66 ± 0.06	5.17 ± 0.56	0.35 ± 0.05	1.17 ± 0.14	0.039 ± 0.005	0.164 ± 0.033	0.36 ± 0.12	0.091 ± 0.019
HS group	0.66 ± 0.08	5.08 ± 0.89	0.35 ± 0.06	1.13 ± 0.15	0.038 ± 0.007	0.208 ± 0.073	0.33 ± 0.10	0.107 ± 0.081
HJM group	0.69 ± 0.10	5.47 ± 0.81	0.35 ± 0.06	1.13 ± 0.14	0.038 ± 0.013	0.218 ± 0.047 **	0.33 ± 0.07	0.091 ± 0.017
Male								
Control group	0.92 ± 0.20	7.29 ± 1.69	0.41 ± 0.11	1.70 ± 0.35	0.031 ± 0.009	0.201 ± 0.103	1.72 ± 0.44	0.35 ± 0.08
HS group	0.92 ± 0.12	7.12 ± 0.91	0.42 ± 0.09	1.69 ± 0.22	0.029 ± 0.007	0.289 ± 0.078	1.78 ± 0.21	0.38 ± 0.05
HJM group	0.98 ± 0.08	7.87 ± 0.97	0.44 ± 0.11	1.77 ± 0.16	0.027 ± 0.007	0.276 ± 0.081	1.80 ± 0.27	0.39 ± 0.04

Compared with the control group, ** *p* < 0.01.

## Data Availability

The data presented in this study are available on request from the corresponding author. The data are not publicly available due to the incompletion of the whole project.
